# Polynorepinephrine nanoparticles activate vascular smooth muscle alpha-1 adrenergic receptors†

**DOI:** 10.1039/d4na00481g

**Published:** 2024-11-18

**Authors:** Vinayaraj Ozhukil Kollath, Vivek Krishna Pulakazhi Venu, Mahmoud Saifeddine, Koichiro Mihara, Simon A. Hirota, Morley D. Hollenberg, Kunal Karan

**Affiliations:** a Department of Chemical and Petroleum Engineering, University of Calgary Calgary AB Canada kkaran@ucalgary.ca; b Department of Physiology & Pharmacology, Inflammation Research Network-Snyder Institute for Chronic Disease, University of Calgary Cumming School of Medicine Calgary AB Canada mhollenb@ucalgary.ca; c Department of Medicine, Inflammation Research Network-Snyder Institute for Chronic Disease, University of Calgary Cumming School of Medicine Calgary AB Canada

## Abstract

Biocompatible polymeric nanoparticles (NPs) as carriers for therapeutic agents with multifunctional activities have received unprecedented attention for a variety of bio-pharmaceutical applications. We describe the synthesis, the fluorescence properties, the bio-compatible nature and the alpha-1 adrenergic receptor bio-activity of engineered quantum dot-like polynorepinephrine (PNE) NPs. The spherical PNE NPs, which are internalized in smooth muscle cells *via* a receptor-selective mechanism, activate alpha-1-adrenoceptors in intact mouse aorta and aorta-derived cultured smooth muscle cells, leading to the activation of calcium signaling/contraction and stimulation of mitogen-activated protein kinase (MAPK), thereby displaying receptor-triggering biological activity, possibly acting both extracellularly and intracellularly. Our data indicate that NPs generated by the polymerization of pharmacologically active compounds like norepinephrine can retain receptor-selective biological activity coupled to inherent fluorescence.

## Abstract 2

Norepinephrine polymerized into quantum dot-like, fluorescent nanoparticles demonstrates receptor-selective, bio-activity by inducing muscle contraction in intact mouse aorta and aorta-derived cultured smooth muscle cells.

## Graphical abstract

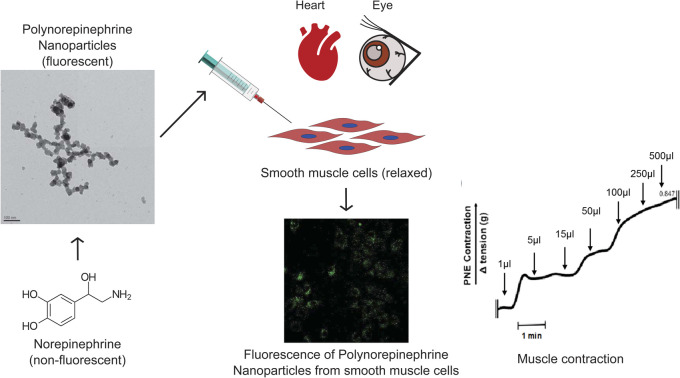

## Introduction

Nanoparticles (NPs) possessing multifunctional properties such as biocompatibility, fluorescence, cell permeability without triggering cytotoxicity, and bio-activity can be an invaluable medical tool with their multi-modality for bio-imaging, diagnosis, and therapy; but such bioactive NPs are rare.^1–4^ This rarity is not surprising because creating multifunctional nanoparticles is a complex process, requiring difficult synthesis steps. For example, in addition to size-control, imparting multi-functionality to nanoparticles often requires tagging with a fluorophore to yield fluorescent properties,^3^ modifying their surface chemistry to obtain a desirable zeta-potential for cell internalization,^5^ and efficiently loading a therapeutic agent (drug or gene) into the particles for drug delivery.^6,7^ Polymerization of catecholamine neurotransmitters, including recent work by the authors,^8–10^ has opened the potential for realizing multifunctional, bio-active nanoparticles.

Akin to dopamine,^11,12^ epinephrine^8^ and norepinephrine^13–16^ can also be polymerized under mild alkaline conditions to create nanoparticles. In a free form, epinephrine and norepinephrine act on both the alpha and beta adrenergic receptors and are used as agents for managing cardiac events and allergic response.^17–20^ Norepinephrine (NE; also termed noradrenaline) is known to cause vascular smooth muscle contraction and other cell responses *via* interaction with both extracellular and intracellular α-1 adrenergic receptors. Thus, poly-NE or PNE nanoparticles lend themselves to potentially retaining bio-functionality in the polymerized form as nanoparticles.

However, compared to polydopamine, there are only a limited number of studies reporting PNE nanoparticles and coatings. A recent review^4^ of polymerized norepinephrine (PNE) studies has identified several knowledge gaps, including: (i) a lack of understanding of the effect on biological systems including living cells and tissues and (ii) a lack of studies on cell toxicity. Pertinent to the current work, a specific unanswered question is: whether or not polymerized NE (PNE) retains any biological functionality *via* its interaction with adrenoceptors either on the cell surface or inside the cells (or both), as compared to its soluble molecular precursor form, NE. Thus, one main focus of our work is to evaluate the biological activity of the PNE nanoparticles in smooth muscle cell and intact tissue assays. It was anticipated that PNE nanoparticles would possess fluorescence,^8^ which would enable the investigation of their uptake mechanism without the need for external fluorophores.

**Scheme 1:**
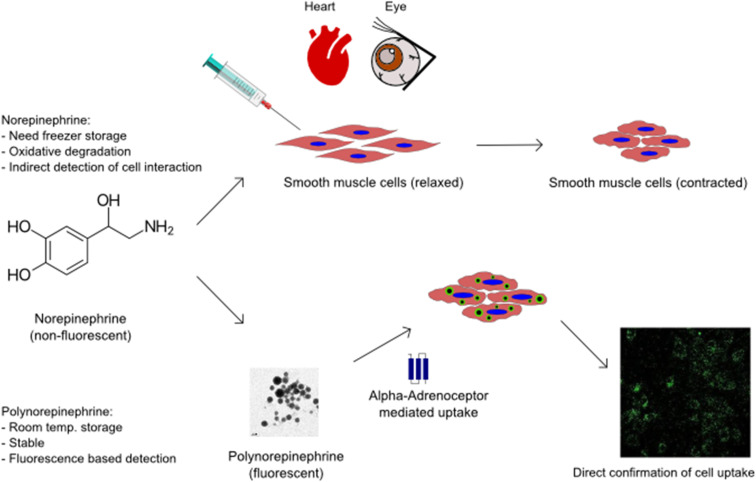
Nanoparticles of polymerised norepinephrine expand the application possibilities of smooth muscle cell based therapeutics.

The present work explores and establishes new functional properties of quantum-dot-like PNE NPs. We first assessed the material properties of the developed NPs and identified their advantages over the soluble precursor, NE. Based on the known bio-functional properties of NE, the biological activities of PNE were assessed in an intact mouse aorta bioassay and in a cell-based assay using primary cultures of mouse aorta-derived smooth muscle cells. The contractile activity of the PNE NPs was evaluated in a vascular aorta ring wire myograph vasoconstriction assay. The PNE NPs were also evaluated for cytotoxicity using the cultured smooth muscle cells and for their ability to stimulate elevation of intracellular calcium and to activate mitogen-activated protein kinase (MAPKinase). The internalization of the PNE-NPs into the cultured smooth muscle cells was also studied to understand the cellular uptake mechanism in comparison to NE. In comparison with soluble NE, polymerised NE nanoparticles with inherent fluorescence will in principle allow for the study of tissue-localized responses to alpha-adrenergic receptor activation (Scheme 1).

## Results & discussion

### PNE-NPs display inherent fluorescence, spherical shape and tunable size

The self-oxidative polymerisation of NE, first reported by Kang *et al.*^13^ and recently by Hong *et al.*,^14^ follows a pathway similar to that of dopamine polymerization.^11,12^ By controlling the nano/microenvironment of such oxidative polymerisation, spherical polydopamine and petal-shaped polyepinephrine nano- and micron-sized particles have been successfully synthesized.^8,21^ Adapting procedures from our previous experiments, spherical PNE NPs were synthesized. We are able to control the size of the spherical PNE NPs by the optimization of the NE : NH_4_OH ratio in the reaction mixture. The resulting NPs were analysed using transmission electron microscopy (TEM) and scanning electron microscopy (SEM) images for size and morphological information.

**Fig. 1:**
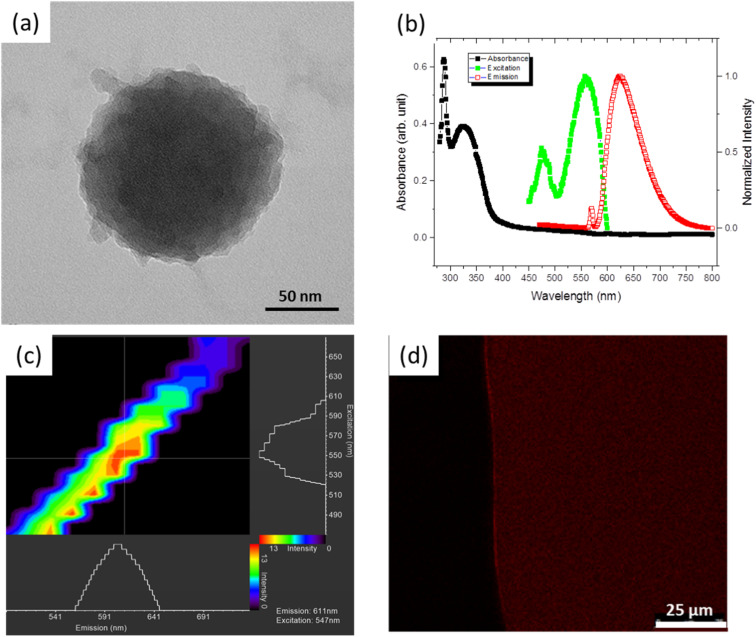
Morphology and fluorescence of PNE NPs. (a) TEM image of the PNE NP synthesized at high (16% v/v) NH_4_OH concentrations; fluorescence characteristics of PNE NPs dispersed in ethanol, characterized by (b) UV-Vis absorption spectroscopy and (c-d) laser scanning confocal microscopy.

Fig. 1a shows the morphology of an ∼100 nm PNE NP prepared at a high NH_4_OH concentration. We were able to vary the size of the spherical PNE NPs controllably by the optimization of the NE : NH_4_OH ratio in the reaction mixture (Fig. S1a and b†). A broad particle size distribution of 20 – 300 nm (Fig. S1c†) was obtained at a lower NE : NH_4_OH (3.4 M) ratio (3.3% v/v); but increasing the NE : NH_4_OH ratio to 16% v/v yielded a narrow size range of 22–30 nm (Fig. S1d†). Although significant aggregation of the NPs upon solvent evaporation was visible in the SEM analysis, the nanoparticles were well dispersed in the aqueous medium and exhibited a zeta potential of about −14 mV.

In addition, Ultraviolet-Visible (UV-Vis) and fluorescence spectroscopies were used to characterize the photo-luminescent properties of the PNE NPs. The absorption spectra in the ultraviolet-to-visible wavelength range and the fluorescence response of PNE NPs dispersed in ethanol are shown in Fig. 1b. The UV-Vis absorption peaks were observed at 288 and 324 nm. When excited at 288 nm, two prominent fluorescence emission peaks at 570 and 622 nm were observed in the wavelength range 400–800 nm. The ∼570 nm peak is from the solvent background. The excitation profile for the emission peak 622 nm was further recorded in the range 450–600 nm, with an excitation maximum at ∼560 nm. The full-width at half maximum (FWHM) of the emission peak of *ca.* 72 nm is at the narrower end of the spectrum of earlier reported carbon quantum dots.^22–26^ The inherent fluorescence properties of PNE NPs were further studied using a laser scanning confocal microscope (LSCM), for the purpose of confocal imaging of the fluorescent NPs. As is evident from Fig. 1c, the NPs respond to excitation wavelengths ranging from 520 to 610 nm with a red-shifted emission wavelength of 550 to 640 nm. This result agrees with the photo-luminescence spectra obtained using the fluorescence spectrometer, considering the laser source used in the LSCM. Fig. 1d shows the confocal fluorescence micrograph of PNE NPs suspended in ethanol, captured immediately after the ethanol evaporation. The result illustrates both the fluorescent and the dispersive properties of the PNE-NPs.

Although PNE NPs dispersed in ethanol showed excellent photoluminescent properties, ethanol is not a suitable solvent for biological applications. Thus, flow cytometry was used to analyse the PNE NP concentration and particle size in an aqueous environment. We performed flow cytometric analysis and found that the PNE NPs could be monitored using optical as well as fluorescent detection channels owing to their physico-chemical properties. The size distribution of the PNE NPs was evaluated using different size standard beads (Fig. S2(a–d)†). Using the standard NFPPS-52-4K (with bead sizes of 0.1–0.3 μm, 0.4–0.6 μm, 0.7–0.9 μm), 2.5 micron nile red beads and the 0.52 micron spheres, PNE-NPs were found to display a spherical shape and a wide range of particle sizes, with a majority under 0.52 μm in diameter, in comparison with the standards used. Also, the fluorescence from the PNE NPs was detected in the flow cytometry experiments using a BV605 channel, which indicated that 27% of the NP population was fluorescently active. The size distribution analyses showed that about 33% of the PNE-NPs in suspension are located in the 0.52 micron sphere gate (Fig. S2a–d panels respectively†). Using medium flow rate (30 μl min^−1^), the total concentration of particles in suspension was determined to be 7.34 × 10^6^ μl^−1^. This estimate allowed us to understand the possible number of particles or varied sizes present in the preparations used for the tissue and cellular bioassays.

### PNE-NPs are biocompatible and non-cytotoxic for cultured smooth muscle cells

**Fig. 2:**
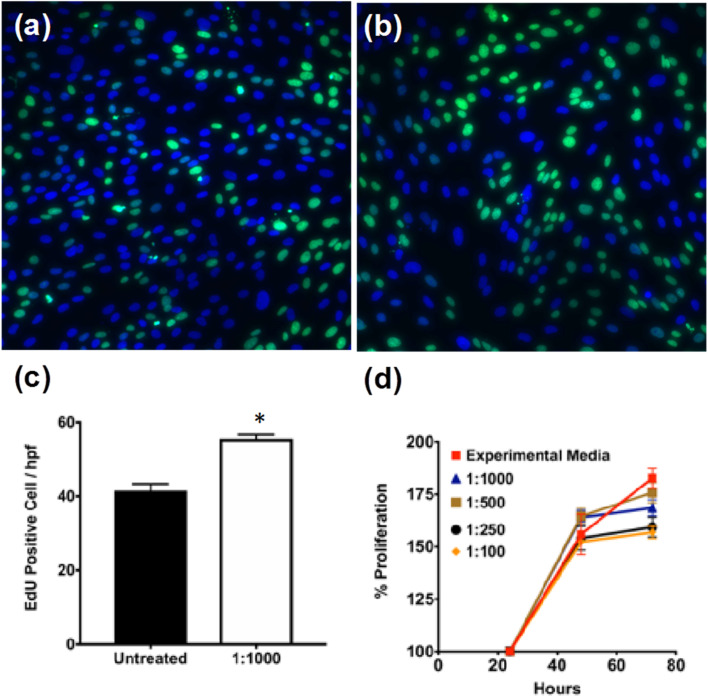
PNE NPs are non-cytotoxic for colon-derived smooth muscle cells. Human colon-derived smooth muscle cells were treated with NPs of 1 : 1000–1 : 100 dilutions from a stock solution of 7.34 × 10^6^ μl^−1^. Fluorescent micrographs of (a) untreated and (b) 1 : 1000 dilution show the EdU incorporation was unaffected, indicating that the PNE nanoparticles are non-cytotoxic as shown by the increase in cell proliferation shown in panels (c) and (d). (d) shows the quantitative measurement of cellular proliferation measured using the WST-1 assay for a period of 72 hours. This result further confirmed that the cells were unaffected for up to 72 hours. Data shown represent the mean ± SD for *n* = 10.

To assess the cytotoxicity of PNE-NPs, cell proliferation was evaluated in several batches of primary cultures of smooth muscle cells of colonic origin. These cells were chosen to evaluate the non-specific PNE-NP toxicity, because unlike primary cultures of smooth muscle cells from mouse aorta (below), the colonic-derived cells did not respond (calcium signaling) to activation by either NE or the PNE-NPs (not shown). Thus, an adrenoceptor-mediated response which might have interfered with the cytotoxicity measurements was avoided. When incubated with different concentrations of PNE-NPs, the colon-derived smooth muscle cells continued to proliferate over 72 hours in all media containing PNE-NPs (Fig. 2a–c). Increasing the concentration of PNE-NPs (decreasing dilutions from 1 in 1000 to 1 in 100) did not affect the cell viability (Fig. 2d). Comparable data showing a lack of cytotoxicity for the nanoparticles were obtained for the primary smooth muscle cell cultures which express functional α-1 adrenergic receptors. These findings affirmed the bio-compatibility and lack of cell toxicity of the PNE-NPs.

### PNE-NPs activate α-1 adrenoreceptors on intact vascular tissue and on aorta-derived primary cultures of smooth muscle cells

**Fig. 3:**
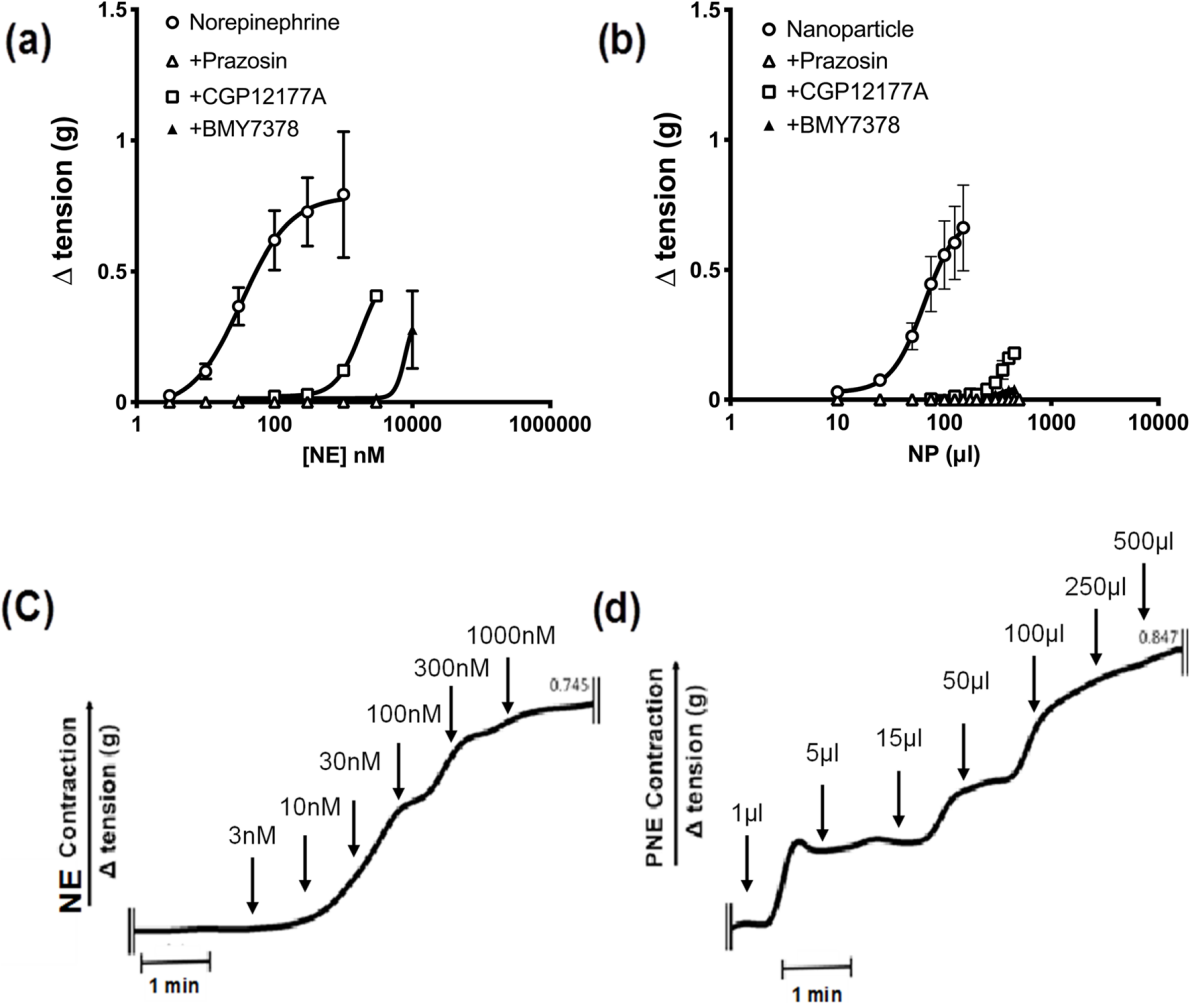
PNE nanoparticles mediate aortic tissue contraction *ex-vivo* through both internal and external α-1 adrenergic receptors. (a and b) Mouse aortic rings upon treatment with increasing concentrations of either NE or one of the PNE nanoparticle preparations showed an increase in vasoconstriction, shown as open circles. Addition of the α-1 antagonist Prazosin (1 μM, open triangles) or CGP12177A (300 μM, open squares) or BMY7378(1–5 μM, closed triangles) inhibited the NE and PNE-NP responses in a pseudocompetitive manner, shifting the concentration–response curves to the right. (c and d) Representative tracings for contractile responses (g tension) caused by the cumulative addition of increasing concentrations of NE (nM) and a second PNE-NP preparation (μl added per 5 ml bath volume). The concentration of PNE-NPs in the suspension added to the organ bath, obtained from flow cytometry, was approximately 7.34 × 10^6^ μl^−1^. *P < 0.05 for differences between control contractions *vs.* tissues treated with Prazosin or other adrenoceptor antagonists. Contractile values are shown as mean ± SD, for *n* = 10.

Next, to identify the bio-functionality of the PNE-NPs, we used wire-myography, as described in detail elsewhere^27–29^ and in more detail in Methods, to assess the vasoconstrictor actions of the PNE-NPs compared to NE itself in isolated mouse aorta rings. As is already known, increasing concentrations of NE cause a concentration-dependent constriction (open circles, Fig. 3a) that was completely blocked by the receptor-selective α-1 adrenoceptor antagonist, prazosin (open triangles, 1 μM). Further, at concentrations that were lower than those required to block contraction completely, the cell-impermeant α-1 adrenoceptor antagonist, CGP12177A (open squares, 300 μM) as well as the cell-permeant α-1 adrenoceptor antagonist, BMY7378 (closed triangles 1–5 μM), both shifted the norepinephrine concentration–effect curve to the right, as would be anticipated for these receptor-selective competitive α-1 adrenoceptor antagonists (Fig. 3a).

The time-frame of response upon adding the PNE-NPs to the tissue bath was comparable to that of adding soluble NE (Fig. 3c and d) The three antagonists similarly blocked the contractile actions of the PNE-NPs (Fig. 3b: shift to the right for CGP12177A and BMY7378; complete blockade for prazosin at 1 μM). Thus, unambiguously the PNE-NPs caused constriction by mimicking the action of NE on the vascular tissue α-1 adrenoceptor. The pharmacologically receptor-selective inhibitors of α-1 adrenergic signaling were all able to inhibit the contractions elicited by the PNE-NPs. Importantly, although 5 to 20 μl of intact PNE-NP suspensions caused a strong contraction, volumes up to 100 μl of nanoparticle-free supernatant from 4 independent PNE-NP preparations did not cause a contractile response in the aorta tissue.

### PNE-NPs elicit a calcium response in cultured vascular smooth muscle cells through an α-1 adrenergic receptor-mediated pathway

After the evaluation of the action of the PNE-NPs on intact aorta vessels and on cultured intestinal smooth muscle cells which are devoid of α-1 adrenergic receptors, their action was assessed using primary cultures of mouse aorta-derived smooth muscle cells, prepared as outlined in Methods. The PNE-NPs triggered an elevation of intracellular calcium in the cultured vascular smooth muscle cells (Fig. 5f, i and S3†); and as for the signal caused by NE, the PNE-NP-triggered increase in intracellular calcium was also blocked by the α-1-adrenoceptor-selective antagonists, CGP12177A and BMY7378 (Fig. 5d, e, g–i). Prazosin also blocked the PNE-NP-stimulated calcium signal, in keeping with its inhibition of PNE-NP-stimulated contraction, shown in Fig. 3b.

There is now unequivocal evidence that a variety of G-protein receptor-coupled agents, including muscarinic,^30,31^ angiotensin II,^32^ endothelin^33^ and adrenergic^34,35^ receptor agonists can act by stimulating intracellularly localized receptors.^36–38^ Since work with the dopamine-derived nanoparticles demonstrated their cellular internalization,^10^ we attempted to ascertain if NE-derived NPs were internalized and might act intracellularly in the primary cultures of aorta-derived smooth muscle cells.

**Fig. 4:**
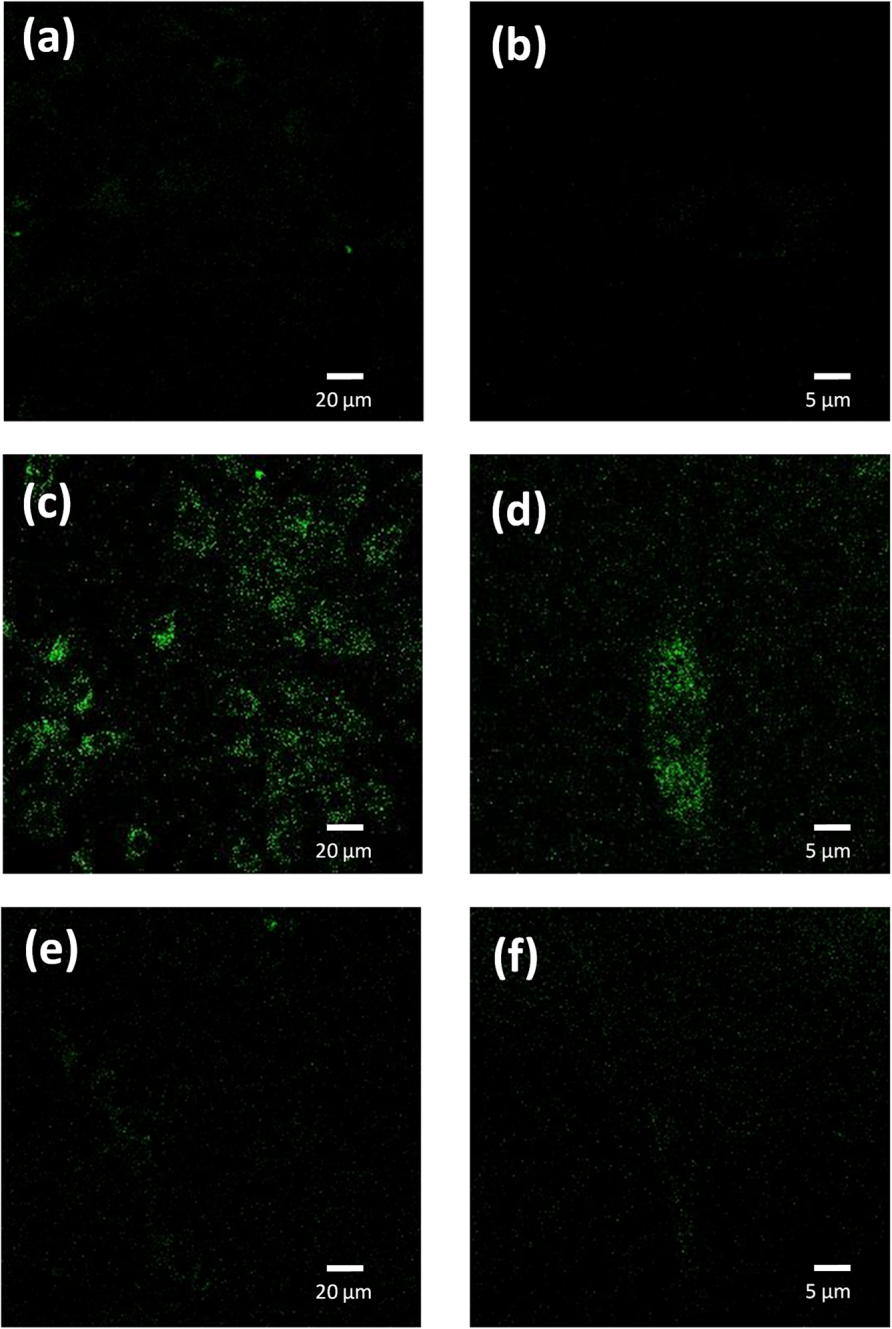
PNE NPs are internalized by smooth muscle cells. Confocal fluorescence micrographs at two imaging magnifications (white bars: 20 μm, left panels; and 5 μm, right panels) show the absence of any background signal in vascular smooth muscle cells (a and b), the uptake of PNE-NPs (PNE) in NP-treated cells (c, d), and the minimal uptake of PNE-NPs in cells pre-treated with the extracellular alpha-adrenoceptor antagonist, CGP1217A (1 μM), prior to the addition of PNE-NPs (e and f).

We used confocal fluorescence imaging to study the internalization of the PNE-NPs. The intrinsic fluorescence of PNE NPs enabled tracking of the uptake of NPs by the vascular smooth muscle cells, consistent with the previous data obtained for the dopamine NPs.^10^ When added to the cultured aorta-derived smooth muscle cultures, apparently internalized fluorescent NPs could be visualized (green fluorescence, Fig. 4, panels c and d). However, upon pre-treatment of the cells with CGP12177A (an extracellular α-1 adrenoceptor inhibitor) for 15 minutes followed by exposure of the cells to the PNE-NPs, the PNE-NPs could not be visualized intracellularly (Fig. 4 panels e and f). This result indicated that the uptake of the NPs was mediated *via* the α-1 adrenoceptor.

**Fig. 5:**
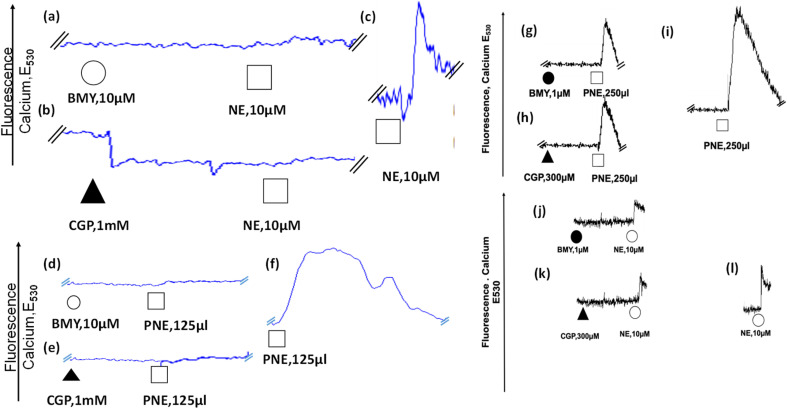
Intracellular calcium signaling caused by norepinephrine (NE) and PNE-NPs (PNE) is blocked by adrenoceptor antagonists that block either the plasma membrane (CGP12177A) or intracellular (BMY7378) receptors. (a) Mouse aorta-derived cultured smooth muscle cells treated with BMY7378 (intracellular antagonist: a, d) or CGP12177A (extracellular antagonist: b, e) prior to the addition of either PNE-NPs (d, e) or NE (a, b) display a reduced intracellular calcium-induced fluorescence signal, compared to the addition of either PNE-NPs or NE alone (c, f). Representative tracings show the reduction in calcium responses at lower concentrations of antagonists (g–l). The PNE NP concentration of the stock suspension used for the additions shown (125 to 250 μl: d, e; g–i) was 7.34 × 10^6^ μl^−1^. Th *E*_530_ calcium signals for panels g and h are shown at the same amplification as for the control signal shown in panel i; and the *E*_530_ calcium signals for panels j and k are shown at the same amplification as for the control signal shown in panel l. The reduced responses in panels g and h are thus directly comparable to the increased signals in panel i; and the reduced responses in panels j and k are also directly comparable to the increased signal in panel l.

To determine the ability of the PNE-NPs to signal from the intracellular compartment, we adopted a strategy using the cell-impermeant α-1 adrenoceptor antagonist, CGP12177A, as well as the cell-permeant α-1 adrenoceptor antagonist, BMY7378. In principle, CGP12177A should block only the extracellular actions of the PNE-NPs and norepinephrine itself; and BMY7378 should block the intracellular signals. Alternatively, prazosin, which acts both extracellularly and intracellularly, should block all actions of both norepinephrine and the PNE-NPs. As shown in Fig. 5, we found that high concentrations of either CGP12177A (1 mM) or BMY 7378 (10 μM) inhibited the cultured vascular smooth muscle cell calcium response to both NE (Fig. 5a and b) and PNE (Fig. 5d and e), compared with the calcium signals observed in control antagonist-untreated cells (Fig. 5c and f). Further, titrating down, lower concentrations of BMY7378 (1 μM) and CGP12177A (300 μM) were also able to inhibit PNE-NP- (Fig. 5g and h) and NE- (Fig. 5j and k) mediated calcium release selectively, compared to their respective controls (Fig. 5i and l). The imaging experiments, as discussed above, showed that the extracellularly acting antagonist, CGP12177A (Fig. 4) blocked the internalization of the PNE nanoparticles. In sum, the PNE-NPs were able to mimic the actions of NE even after polymerisation. These data further reinforced the point, both pharmacologically and through imaging, that the PNE-NPs can stimulate cell signaling and can be internalized in a receptor dependent manner. As a further test to see if the PNE-NPs would reliably mimic the actions of NE, we evaluated the ability of the PNE-NPs to activate the MAPKinase signaling pathway in the smooth muscle cell cultures.

### PNE-NPs mediate MAPK signaling through the external α-1 adrenergic receptor mediated pathway in vascular smooth muscle cells

**Fig. 6:**
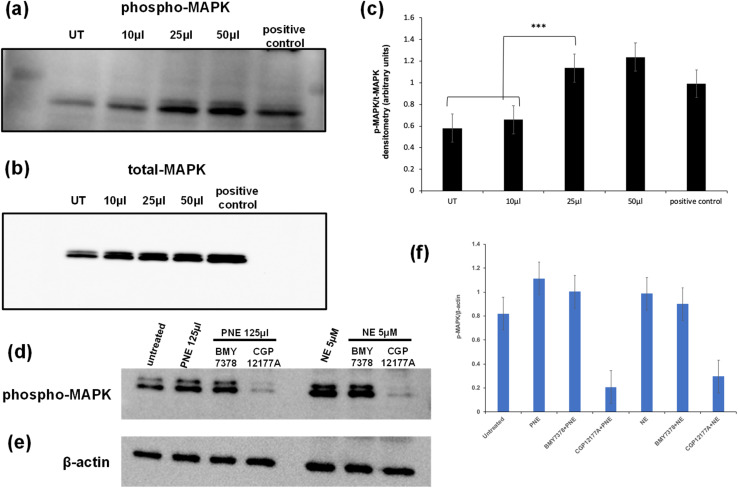
Activation of MAPKinase in primary cultures of murine aorta-derived smooth muscle cells by PNEs and NE: block by an extracellular alpha adrenoceptor antagonist, CGP12177A, but not by an intracellular receptor antagonist, BMY7378. Cultured aorta-derived vascular smooth cells treated with increasing concentrations of PNE NPs (10 to 50 μl of a stock solution of 7.34 × 10^6^ NPs per μl added to the 1 ml cell medium) display an increased abundance of activated phospho-MAPK (panel a), normalized to the total abundance of MAPKinase in the cell blots (panel B). Representative blots are shown for the phosphorylation of MAPK (a) and total MAPK (b). The densitometry data from blot imaging, normalized to the total abundance of MAPK in each of three independent blots, are shown by the histograms in panel c. The involvement of internal and external adrenergic receptors was evaluated by pre-treating with CGP12177A (extracellular antagonist: 300 μM) or BMY7378 (intracellular antagonist: 1 μM) for 10 minutes, followed by addition of PNE-NPs or NE for 5 minutes is shown by the representative phospho-MAPK blots (panel d), normalized to the beta-actin signal (panel e). The averaged densitometry data, normalized to the total abundance of beta-actin in each western blot lane (mean ± SD, from *n* = 3–4 independent experiments), are shown by the histograms in panel f. The PNE NP concentration of the stock solution used for adding the PNE NP nanoparticles (10 to 125 μl added to each 1 ml culture well) was 7.34 × 10^6^ μl^−1^. Data are shown for the histograms represent the mean ± SD, *n* = 3–4 independent experiments.

In addition to elevating intracellular calcium *via* activation of Gq, the stimulation of the α-1 adrenoceptor can also cause the activation of MAPKinase. In principle, activation of this signal pathway could be caused either *via* the stimulation of a cell surface receptor or possibly *via* an intracellular endosomal signaling process. To evaluate the action of the PNE-NPs in this regard, cells were treated with increasing volumes of PNE-NPs (7.34 × 10^9^ ml^−1^) and the resulting activation of MAPKinase was assessed as shown by the western blot analysis of phospho-MAPKinase shown in Fig. 6. We observed an increase in MAPKinase activation/phosphorylation upon the addition of 25–50 μl of PNE-NPs that maximally was equivalent to or slightly greater than the addition of 10 μM norepinephrine (histograms, Fig. 6c). These data allowed us to confirm the functional activity of the PNE-NPs to mimic the action of NE in cultured vascular smooth muscle cells in terms of both the calcium and MAPKinase signaling pathways.

We then asked: do PNE NPs function through an intracellular or extracellular interaction with the α-1 adrenergic receptor, or possibly in conjunction with receptors localized in both the cellular compartments? To answer this question, we pretreated vascular smooth muscle cells with either CGP12177A (1 mM) that primarily targets the extracellular adrenoceptor or BMY7378 (10 μM) that targets the intracellular receptors, followed by the treatment with either PNE-NPs (125 μl ml^−1^; concentration = 7.34 × 10^9^ ml^−1^) or NE itself. We used relatively high concentrations of the inhibitors to see if the MAPKinase signal could be completely abrogated. The inhibitors were used at concentrations that reduced the calcium signal by about 80% (Fig. 5 g, h; j and k). We found that treatment with the extracellular antagonist, CGP12177A, reduced the MAPKinase activation signal by more than 50% for stimulation by either the PNE-NPs or by NE itself, whereas the antagonist believed to act mainly intracellularly (BMY7378) did not cause a statistically significant reduction in MAPK phosphorylation compared to the controls for either PNE-NPs or NE (Fig. 6d–f). In contrast, similar levels of reduction in calcium signaling were caused by the two antagonists used at the same concentrations for cell activation by either the PNE-NPs or by NE (Fig. 5 g, h; j and k). These data not only indicated that PNE-NPs mimic the action of NE in terms of MAPKinase activation, but also suggested that for both the PNE-NPs and NE, a large portion of the MAPKinase signaling is through cell surface α-1 adrenergic receptors.

## Conclusions

In summary, the cross-correlated dataset of the present study has established that the polymerized PNE-NPs, which could be visualized by their fluorescent properties when internalized in cells, mimic the ability of free norepinephrine molecules to activate the cell-localized surface α-1 adrenergic receptor. The data support the conclusion that the PNE-NPs can act on responsive cells in a highly localized manner. Thus, in principle, the NPs would be able to identify NE-responsive cells in a complex tissue and to activate α-1 adrenergic receptor-expressing cells selectively upon local administration *in vivo*. It is notable that the PNE-NPs exhibit actions consistent with the observations by Venter and colleagues who demonstrated the ability of glass bead-bound isoproterenol to cause adrenergic responses in chicken and dog heart tissues (Venter *et al.*, 1972). Furthermore, the size-controlled, intrinsically fluorescent, PNE-NPs that can be synthesized by a facile, one-pot method may prove of value in tissue settings to activate and, *via* internalization, identify discrete NE-responsive cells that possess α-1 adrenergic receptors.

## Resource availability

### Lead Contact

Further information and requests for nanoparticle synthesis and physico-chemical characterization should be directed to and will be fulfilled by the Lead Contact, Prof. Kunal Karan (kkaran@ucalgary.ca). For queries and information related to bio-activity assessments, requests should be directed to Prof. Morley Hollenberg (mhollenb@ucalgary.ca)

### Materials availability

This study did not generate new unique reagents.

## Data availability

The published article includes all datasets generated or analyzed during this study. The data supporting this article have been included as part of the ESI.† Blot data have been provided as a separate file.

## Author contributions

VOK, KK designed and performed PNE-NP synthesis and material characterization. VKPV, MS and MH conceived, designed and in part conducted all cell assays, tissue bioassays, flow cytometry, western blotting, and calcium imaging assays using the PNE NPs. They also analysed and interpreted all data. KM performed imaging experiments. VOK, VKPV, KK, SAH and MH – analysed and interpreted the data and wrote the manuscript.

## Conflicts of interest

The authors declare no conflict of interests.

## Supplementary Material

NA-OLF-D4NA00481G-s001
