# Supplementary material for: Polynorepinephrine nanoparticles activate vascular smooth muscle alpha-1 adrenergic receptors
Source: Nanoscale Adv. 2024 Nov 18;7(3):830–9. doi: 10.1039/d4na00481g (PMC11647915; doi:10.1039/d4na00481g)
Supplement: NA-007-D4NA00481G-s001 [file NA-007-D4NA00481G-s001.pdf]

Supplementary Information (SI) for Nanoscale Advances.
This journal is © The Royal Society of Chemistry 2024

Supplementary information

Polynorepinephrine nanoparticles activate vascular smooth muscle alpha-1 adrenergic receptors

Vinayaraj Ozhukil Kollath[1 ‡], Vivek Krishna Pulakazhi Venu[2 ‡], Mahmoud Saifeddine[2], Koichiro Mihara[2], Simon A. Hirota[2], Morley D. Hollenberg[2,3 *], Kunal Karan[1 *]

[1]Department of Chemical and Petroleum Engineering, University of Calgary, Calgary, AB, Canada

[2]Department of Physiology & Pharmacology, Inflammation Research Network-Snyder Institute for Chronic Disease, University of Calgary Cumming School of Medicine, Calgary, AB, Canada

[3]Department of medicine, Inflammation Research Network-Snyder Institute for Chronic Disease, University of Calgary Cumming School of Medicine, Calgary, AB, Canada

‡ - Authors made equal contribution

*Corresponding Authors

Prof. Kunal Karan

Department of Chemical and Petroleum Engineering,

University of Calgary, Calgary, AB, Canada

Email: kkaran@ucalgary.ca

and

Prof. Morley D. Hollenberg

Department of Physiology & Pharmacology, Inflammation Research Network-Snyder Institute for Chronic Disease, University of Calgary Cumming School of Medicine, Calgary, AB, Canada

Email: mhollenb@ucalgary.ca

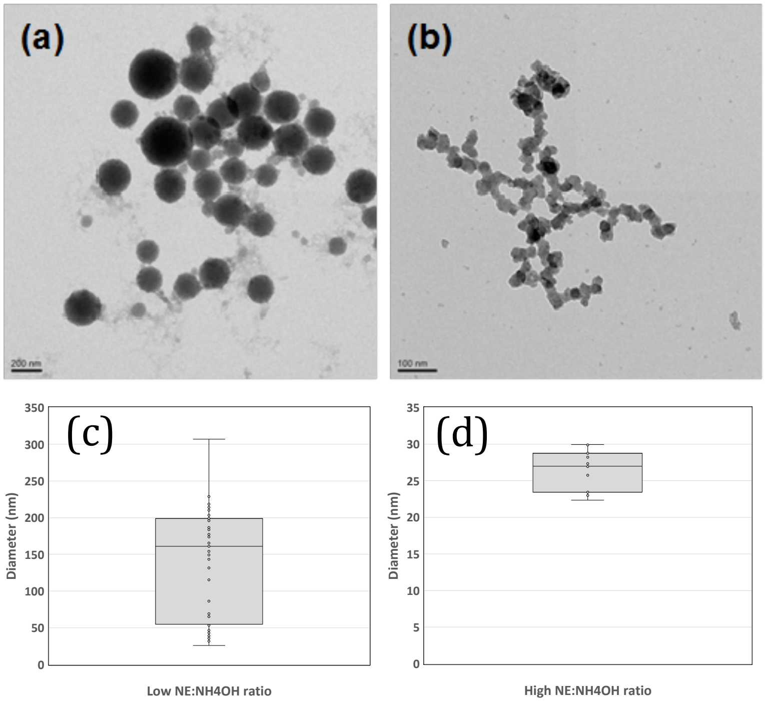

Figure S1: (a, b) Transmission electron micrographs showing aggregation and morphology of PNE NPs; (c) and (d) box plot of particle size of PNE-NPs visualized from TEM image (a) and (b) with ncount of 49 and 14, respectively.

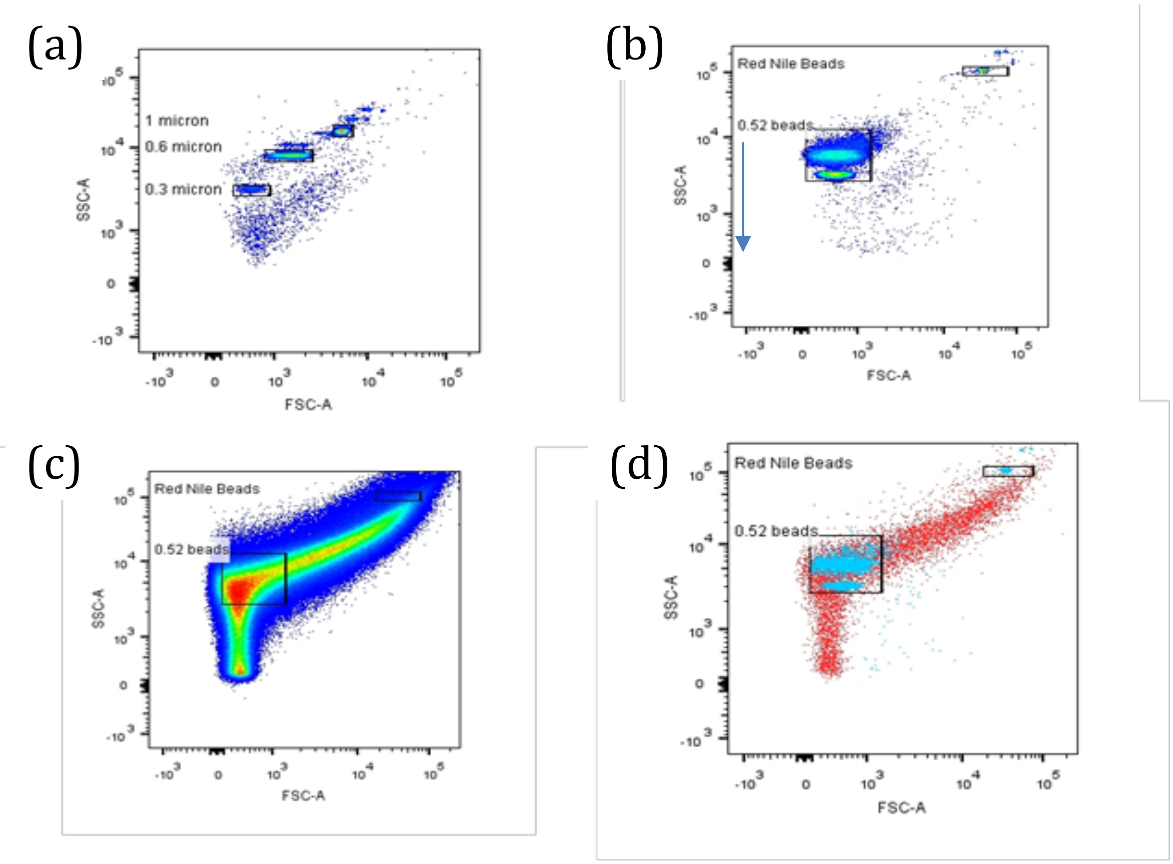

Figure S2: Flow cytometry results are shown in (a-d). (a) NFPPS-52-4k standard beads, (b) Red Nile beads and 0.52 μm microbeads (c) PNE-NPs and (d) shows overlapping of PNE-NP with the 3 standards.

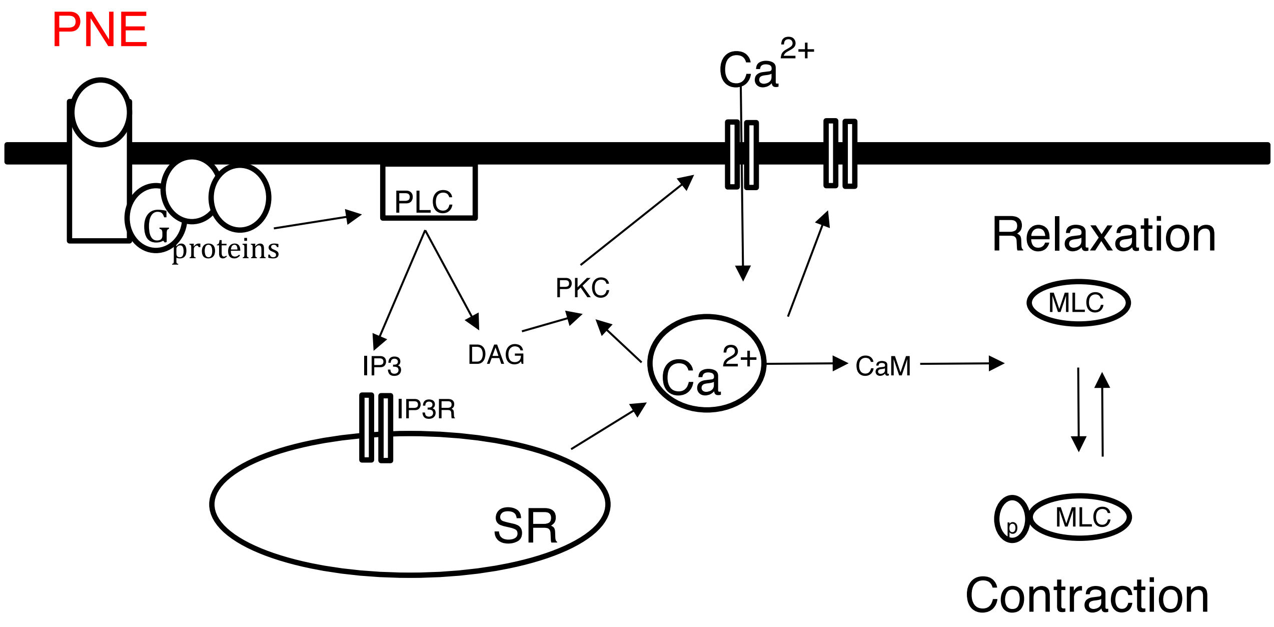

Figure S3: Schematic representation of Ca2+ assay and proposed mechanism of PNE-NP function.

Materials & Methods

Norepinephrine hydrochloride, Prazosin hydrochloride (Sigma-Aldrich, MO, USA), anhydrous ethanol (Commercial Alcohols, ON, Canada), MAPK inhibitor (LY294002 hydrochloride, Tocris, Bristol, UK) and Ammonium hydroxide (50 % v/v aqueous solution, Alfa Aesar, MA, USA) were used without further purification. For all syntheses, high purity deionized water (DI water; 18 MΩ, Milli-Q Advantage A10, Millipore SAS, France) was used.

Synthesis of PNE NPs: The NP synthesis was adapted from our previously reported method.[1] In a typical batch preparation of nanoparticles, 53 mg Norepinephrine hydrochloride was added to a total volume of 15 ml mixture of 33.3 % v/v of absolute ethanol, 63.3 % v/v DI water and 3.3 % v/v NH_4OH aqueous solution (3.4 m) and stirred for 2 h at room temperature (RT). A color change from clear to brown indicated the oxidative polymerization of norepinephrine to polynorepinephrine (PNE). The resulting suspension was then dialyzed extensively against distilled deionized water at room temperature using Spectra/Por dialysis membrane tubing (12-14 kD cutoff, S432706 Fisher Scientific) until the dialysate became neutral (pH, 7.4±0.3). The dialyzed NP suspension was stored at RT and used without further processing. Concentration of NPs in the resulting suspension was quantified using flow cytometry as described below.

Physicochemical characterization of PNE nanoparticles: The morphology of PNE nanoparticles (NPs) was characterized using scanning electron microscopy (SEM; XL30, FEI Corp.) and transmission electron microscopy (TEM; Tecnai F20, FEI Corp.). Particle size analysis of PNE NPs was conducted using ImageJ v1.46 software. Absorption and photo luminescence spectra of PNE NPs dispersed in ethanol were studied using a Varian CARY 300 Bio UV-Visible spectrophotometer and a Horiba Jobin Yvon Fluoromax-4 spectroscope, respectively. The path length of the cuvette used was 10 mm in both cases. For a quantum yield (QY) calculation, integrated fluorescence intensities of PNE NPs dispersed in ethanol were plotted versus the respective absorbance values (all absorbance under 0.1 at 320 nm). Then, the slope of the curve was used to calculate the QY of PNE with reference to the known values of a quinine sulfate standard (both excited at 320 nm) as reported by Pan *et al.*[2] Laser scanning confocal microscopy (LSCM) was performed using a Leica SP8 X inverted confocal microscope equipped with a White Light Laser (470-670 nm). The excitation wavelength was increased from 470 nm at a 10 nm step size until 670 nm, while the detection range was set to 480 – 750 nm. For imaging the PNE, NPs were first dispersed in ethanol and homogenized by 10 min ultrasonication (Branson 2800 series, Branson Ultrasonics Corp., CT, USA). The homogenized dispersion was dropped on a glass coverslip, dried and placed under the microscope for imaging using a 63× oil immersion objective.

Flow Cytometry of the NPs was carried out at a dilution of 1 in 10000 along with standard reference beads, in a BD FACSCanto 10 color (San Jose, USA) instrument. Carboxylate 0.52 µm Polybead® microspheres (Cat #09836, Polysciences Inc., USA) and 2.49 µm Nile red reference beads (BD, San Jose, USA) were used as reference standards. A drop of Nile red polybeads or a drop of both sets of beads were added to a polystyrene tube containing 0.2 micron filtered Millipore water along with the diluted norepinephrine nanoparticle sample. In addition, a Nano Fluorescent Size Standard Kit (cat # NFPPS-52-4K, Yellow, Flow Cytometry Grade, Spherotech, Lake Forest, USA) was used as a quality control.

Tissue Isolation and wire-myography measurements of PNE-NP-increases in vascular tension: All experiments were done using aorta tissues from 8-12 week old male wild-type C57BL/6 mice (N= 5 mice) (20 to 25 g). Bioassay protocols were approved by the University of Calgary Animal Care Committee and are described elsewhere.[3,4] Prior to euthanasia, animals were heparinized (100 µl containing 1000 IU/ml of heparin administered intraperitoneally) and then euthanized 10 min later by isoflurane. Isometric tension studies using a Mulvany–Halpern myograph were performed essentially as described previously.[3] In brief, descending aorta tissue was cut into 2 mm long and 2 mm diameter rings and mounted in a wire-myograph (610 multimyograph system coupled to chart5 system software, AD instruments,

Colorado Springs, CO, USA). All assays were performed at 37 °C using isotonic Kreb's buffer, pH 7.4, aerated with 5 % CO_2 in air. Tissues were washed 4X after reaching an equilibrium tension in response to either the PNE-NPs or NE and were allowed to re-equilibrate in bioassay buffer for 20 minutes prior to the next addition of agonists to the organ bath. After validating the responsiveness of the tissues to KCl and PE-stimulated constriction and acetylcholine-mediated endothelium-dependent vasorelaxation, increasing amounts of the the PNE-NP preparation (1 µl to 1000 µl; Concentration = 7.34×10^6 μl^{-1}) were added to the organ bath, and the resulting contractile responses were compared to those stimulated by increasing concentrations of NE. The recordings were analyzed using Lab Chart (ADI Instruments). To assess the action of receptor-selective alpha-1-adrenoceptor antagonists (Prazosin 1 µm, CGP21277A 300 µm, BMY7378 1 µm). All antagonists bought from Sigma Aldrich. Increasing concentrations of antagonist were added to the organ bath 20 minutes before the addition of a contractile concentration of either norepinephrine (1 µm) or PNE-NPs.

Primary cultures of mouse aorta-derived vascular smooth muscle cells: Isolation of vascular smooth muscle cells from mouse aorta was performed as described before.[5] Animals were euthanized as described in the above section and isolated aortic segments were placed in one well of a 6-well plate; and allowed to adhere for a minute. The tissues was covered just enough with DMEM/F12 media containing 5 % FBS and plasmocin. Care was taken not to move the tissues. The cells sprouting by day 10 were cultured and propagated using the same medium. Cells were used for assays after passage 3. The smooth muscle phenotype of the cells was confirmed using western blot analysis for Calponin, a specific smooth muscle cell marker.

5-ethynyl-2'-deoxyuridine (EdU) -Cell Proliferation assay: The rationale for using Human colonic smooth muscle cells is to know any impact on cells expressing low or no α-1 adrenergic receptors (Catalogue number, CSC-7803W, Creative Bioarray, NY, USA). The cells were seeded onto a 96-well plate at a density of 5×10^3 cells per well. The cells were allowed to attach overnight. Cells were treated with nanoparticles (NP) at a concentration of 1:1000 – 1:100 dilution (NP suspension:Media). Water soluble tetrazolium salt (WST-1) assay was performed as per manufacturer's instruction (Sigma Aldrich, USA). In brief, 10 µl of WST-1 solution was added per well and the plates were incubated at 37 °C under an atmosphere of 5 % CO_2 in room air for 90 minutes. The resulting change in absorbance was read using a spectrophotometer (SpectraMax plus, Molecular devices, CA, USA) at a wavelength of 490 nm. EdU incorporation assay was performed as per manufacturer's instruction (Invitrogen, Carlsbad, CA, USA). In brief, cells were incubated with different dilutions of PNE NP in optiMEM media supplemented with 10 µM EdU for 24 h. Cells were then washed in isotonic Phosphate Buffered Saline pH 7.4 and fixed using 3.7 % formaldehyde. Cells were then permeabilized using 0.5 % Triton X-100 in PBS and stained for EdU using components provided in the kit. The cells were then visualized for Hoechst (under DAPI filter; blue fluorescence) and EdU (under GFP filter; green fluorescence) using Fluorescence microscopy (Nikon Eclipse Ti-U). The percentage of EdU-positive cells (green fluorescence - representative of replication) was calculated via image analysis using the software ImageJ (v1.46).

Measurement of norepinephrine and PNE-NP-stimulated calcium signalling in cultured vascular smooth muscle cells: Measurements of phenylephrine and PNE-NP-stimulated calcium signaling in cultured vascular smooth muscle cells were done essentially as described previously using cultured human kidney-derived HEK cells.[6] In brief, primary cultures of mouse aorta-derived were grown to approximately 80 % confluency in DMEM/F12 media in 25 cm^2 T25 flasks. Cells were detached in enzyme-free isotonic phosphate-buffered saline supplemented with 10 mM EDTA 10 mM and were then re-suspended in EDTA-free Hanks buffered isotonic saline pH 7.4 containing 1 mm Calcium and / or 1 mm magnesium chloride. Vascular smooth muscle Cells from a single T75 flask were harvested by centrifugation (1000 rpm - for 5minutes at room temperature) in 15 ml centrifuge tubes and re-suspended in 1 ml of Fluo-4-

AM 'no-wash' calcium indicator dye (Thermo Fisher Scientific, USA.). Calcium indicator dye uptake was allowed to proceed with shaking for 40 min at RT. Fluorescence measurements reflecting the elevation of intracellular calcium were conducted using a PerkinElmer Fluorescence spectrometer (PerkinElmer instrument, Shelton, CT, USA) with an excitation wavelength of 480 nm and emission of 530 nm. Cells (4×10^5) were maintained in suspension in a stirred plastic cuvette and PNE-NPs (125 µl ml^{-1} corresponding to 9.18×10^8 NPs ml^{-1}) or norepinephrine (5 µm) were added to assess agonist-induced changes in fluorescence. As for the intact tissue bioassay, to assess the action of receptor-selective alpha-1-adrenoceptor antagonists on agonist-stimulated calcium signaling, prazosin, CGP12177A or BMY7378 were added to the cell suspensions 10 minutes prior to the addition of either norepinephrine or the PNE-NPs.

Confocal fluorescence imaging to visualize PNE-NPs internalization in vascular smooth muscle cells: Isolated VSMC's were grown to 80 % confluence in T25 flasks in 25 mm glucose DMEM/10 % fetal bovine serum. Cells were lifted using isotonic 1mM EDTA-containing phosphate buffer saline lacking calcium and magnesium (PBS) and finally re-plated in 35 mm glass bottom cell culture dishes (MatTek Corporation, Ashland MA USA) in a volume of DMEM high glucose with 5 % fetal bovine serum. After 2 days, 125 µl·ml^{-1} volume of PNE-NPs were added to the cell monolayers in a total volume of 1ml either without or with the prior addition of 300 µM CGP12177A for 10 minutes. After 10 minutes of incubation with the PNE-NPs, the cell monolayers were washed 3 times with 1 ml isotonic PBS then fixed with 1 ml of 10 % formalin for 10 minutes. The fixed cells were washed with PBS for 5 times and were then visualized using confocal fluorescence microscopy to monitor NP internalization. Images were collected on a Zeiss LSM-510 Meta inverted microscope using either a 40× water immersion or a 63× oil 1.4NA oil immersion lens. The PNE-NP particle fluorescence (Figure 4) was visualized using a 515/50 nm band-pass filter upon excitation with an argon laser at 488 nm. Image acquisition was performed with identical gain, contrast, laser excitation, pinhole aperture, and laser scanning speed for all images shown in the figures wherein scale bars are included.

Western blot detection of agonist-activation of MAPKinase in primary cultures of aorta-derived smooth muscle cells: The aorta-derived smooth muscle cells were grown to 80 % confluency using smooth muscle cell growth medium (DMEM low glucose media with 5 % FBS and antibiotics penicillin and streptomycin 1 U·ml^{-1}) in 24-multiwell plates of 15.6 mm diameter/well. Monolayers were then treated for 5minutes with norepinephrine 1 µm or PNE-NPs of increasing volume (10-25 µl; Concentration = 7.34×10^6 $µl^{-1}$) added to a total volume of 1 ml. At 5 min after agonist addition, the medium was aspirated followed by the addition of ice-cold lysis buffer and then lysed and homogenized using 1 mL of ice-cold proteinase-inhibitor-containing NP40-supplemented lysis buffer with the following composition: 20 mm Tris-HCl, pH 7.5, 100 mm NaCl, 10 mm $MgCl_2$, 1 mm EDTA, 1 mm EGTA 0.5% NP40, 2.5 mm sodium pyrophosphate, 1 mm b-glycerophosphate, 1 mm Na_3VO_4, 25 mm NaF, 1 mg ml^{-1} leupeptin, 1 mg ml^{-1} aprotinin, 1 mM phenylmethylsulfonyl fluoride, and 1 mm dithiothreitol. To 1 ml of lysis buffer 10 µl of proteinase inhibitor cocktail was also added (calbiochem cocktail set III, EDTA-free cat. no. 539134). Equivalent amounts of protein (measured by precision red reagent, Cytoskeleton. Inc.) from each cell monolayer extract were heat-denatured at 92 °C for 6 min in denaturing Laemmli buffer and resolved on 4–20 % gradient Novex Tris-Glycine gels (ThermoFisher) run at 120 V for 2 h. Transfer of proteins onto PVDF membrane was done using a semi-dry method as described elsewhere.[7] The resolved proteins were transferred to PVDF membrane, blocked for 1 h at RT in PBST buffer [Phosphate-buffered isotonic saline supplemented with 0.1 % (v/v) Tween-20] containing 1% ECL Advance Blocking Agent (GE Healthcare, Waukesha, WI). The abundance of both phospho-MAPK and total-MAPK were quantified using appropriate antibodies (Cat # 9106L and 4695S, Cell signaling technologies) conditions for primary antibodies as described previously.[7] Upon incubation with respective secondary antibodies for 1 h. the membranes were washed for 5 min 3 times using PBST. After washing the membrane with PBST, the peroxidase activity was

detected with the chemi-luminescence reagent ECL-Advance (GE Healthcare, Waukesha, WI) on a Biorad reader. Band intensities representing p-MAPK/total-MAPK were quantified using the Image J quantification software and normalized to beta-actin. The experiments were repeated a minimum of 3-times and the signals for phospho-MAPKinase was normalized to the signal for total MAPKinase detected in the blot.

Statistical Analysis: The data and statistical analysis were performed using GraphPad Prism software. The mean log concentration–response curves to agonists were analyzed by fitting data to a four-parameter logistic equation using non-linear regression (pEC50 log(agonist) vs response – variable slope (four parameters)) (GraphPad Prism). Error bars smaller than the symbols shown in the figures are not visible. Statistical comparisons between data points were made using either Student's unpaired t-test when comparing two groups or a one-way ANOVA, with Tukey's multiple comparison test when comparing multiple groups. In all cases, a significant difference was considered at $P < 0.05$.

References

[1] V. Ozhukil Kollath, M. Derakhshandeh, F. D. Mayer, T. Mudigonda, M. N. Islam, M. Trifkovic, K. Karan, RSC Adv. 2018, 8, 31967.

[2] D. Pan, J. Zhang, Z. Li, C. Wu, X. Yan, M. Wu, Chem. Commun. 2010, 46, 3681.

[3] V. K. Pulakazhi Venu, M. Saifeddine, K. Mihara, M. El-Daly, D. Belke, J. L. E. Dean, E. R. O'Brien, S. A. Hirota, M. D. Hollenberg, Br. J. Pharmacol. 2018, 175, 2063.

[4] M. El-Daly, V. K. Pulakazhi Venu, M. Saifeddine, K. Mihara, S. Kang, P. W. M. Fedak, L. A. Alston, S. A. Hirota, H. Ding, C. R. Triggle, M. D. Hollenberg, Vascul. Pharmacol. 2018, 109, 56.

[5] V. K. P. Venu, P. Uboldi, A. Dhyani, A. Patrini, R. Baetta, N. Ferri, A. Corsini, A. F. Muro, A. L. Catapano, G. D. Norata, Thromb. Haemost. 2015, 114, 186.

[6] A. Kawabata, M. Saifeddine, B. Al-Ani, L. Leblond, M. D. Hollenberg, J. Pharmacol. Exp. Ther. 1999, 288, 358.

[7] K. Mihara, R. Ramachandran, B. Renaux, M. Saifeddine, M. D. Hollenberg, J. Biol. Chem. 2013, 288, 32979.
